# Genome Sequence of the Probiotic Strain *Lactobacillus rhamnosus* (Formerly *Lactobacillus casei*) LOCK908

**DOI:** 10.1128/genomeA.00120-14

**Published:** 2014-02-20

**Authors:** Anna Koryszewska-Bagińska, Jacek Bardowski, Tamara Aleksandrzak-Piekarczyk

**Affiliations:** Institute of Biochemistry and Biophysics, Polish Academy of Sciences, Warsaw, Poland

## Abstract

*Lactobacillus rhamnosus* LOCK908, a patented probiotic strain (Polish patent no. 209987), was isolated from the feces of a healthy 6-year-old girl. Here, we present the complete genome sequence of LOCK908 and identify genes likely to be involved in the biosynthesis of exopolysaccharides (EPSs).

## GENOME ANNOUNCEMENT

*Lactobacillus rhamnosus* LOCK908 (formerly *Lactobacillus casei* LOCK908) was obtained from the Pure Culture Collection of the Technical University of Lódz (Lódz, Poland). To verify the identity of strain LOCK908, sequence analysis and comparisons of the 16S rRNA, *rpoA*, and *pheS* genes were performed, which indicated that LOCK908 belongs to the *L. rhamnosus* species (1).

Previous *in vitro* tests conducted on *L. rhamnosus* LOCK908 revealed the strain to be resistant to gastric acids and bile salts, have antagonistic activity against pathogenic bacteria (2, 3), and have an ability to adhere to the Caco-2 epithelial cell line (4). Moreover, it has been proven by *in vivo* experiments that the mixture of *L. rhamnosus* LOCK908, *L. rhamnosus* LOCK900 (5), and *L. casei* LOCK919 (6) strains modulates the immune system by inducing T_h_1 and regulatory cytokine production and by suppressing the proallergic response (7).

The genomic DNA was extracted using a genomic mini purification kit (A&A Biotechnology). Genomic libraries containing shotgun and 8-kb inserts were constructed, and high-quality reads totaling 155 Mbp were generated by using the GS FLX Titanium pyrosequencing system (Roche). Sequence assembly was performed using Newbler Assembler version 2.4 software (Roche). Contig alignment was performed using SeqMan software from the Lasergene package (DNAStar). To further improve consensus quality, a sequencing run on the Illumina system HiScanSQ was performed, giving 177-fold final coverage of the genome. Functional annotation of genes and rRNA was performed using the RAST annotation server (http:/rast.nmpdr.org/) (8) and checked by BLAST analysis (9) when needed. tRNAs were identified with tRNAscan-SE (10).

The complete genome of *L. rhamnosus* LOCK908 contains a single circular chromosome of 2,990,900 bp, with an overall G+C content of 46.8%. No plasmids were detected in the sequenced DNA of LOCK908. There are 2,931 coding sequences (CDSs) and 60 tRNAs. Ribosomal operons coding for 5S, 16S, and 23S rRNA are present in 5 copies each. The genome sequence of LOCK908 contains 313 subsystems, according to the RAST server. Approximately 32% of the total CDSs were annotated as encoding hypothetical proteins with unknown function.

A comparison of the LOCK908 genome with that of LOCK900 (5), another genome of *L. rhamnosus* sequenced by our group, reveals the former to be larger and therefore to contain an appropriately larger number of protein-coding genes. In particular, the numbers of genes distributed in the categories of cell wall and capsule, DNA metabolism and phages, prophages, and transposable elements are increased in the LOCK908 genome compared to that of LOCK900.

The most significant feature of LOCK908 is its high exopolysaccharide (EPS) productivity, which is relatively common for probiotic *L. rhamnosus* strains (11) , and it has also been observed in our research (our unpublished data). Indeed, EPS-related genes involved in the pathway of capsular and extracellular polysaccharides were identified from genome information. In particular, an 18-kb gene cluster (LOCK908_2101 to LOCK908_2117), which contains 17 EPS-related genes, with a genetic organization and structure typical in the biosynthesis of EPS, was found in the chromosome. In addition, several genes from another cluster (LOCK908_2053 to LOCK908_2071) might be involved in the regulation, chain length determination, biosynthesis of the repeating unit, polymerization, and export of the EPS (12).

### Nucleotide sequence accession number.

The genome information for the chromosome of *L. rhamnosus* LOCK908 has been deposited in the GenBank database with the accession no. CP005485.
